# Effectiveness of a health-social partnership program for discharged non-frail older adults: a pilot study

**DOI:** 10.1186/s12877-020-01722-5

**Published:** 2020-09-10

**Authors:** Arkers Kwan Ching WONG, Frances Kam Yuet WONG, Jenny Sau Chun NGAI, Shirley Yu Kan HUNG, Wah Chun LI

**Affiliations:** 1 School of Nursing, The Hong Kong Polytechnic University, Hung Hom, Kowloon, Hong Kong; 2 Queen Elizabeth Hospital, Kowloon Central Cluster, Hong Kong Hospital Authority, Kowloon, Hong Kong

**Keywords:** Self-care, Non-frail, Health-social partnership, Community-dwelling older adults

## Abstract

**Background:**

Previous studies supporting discharged patients are hospital-based which admission criteria tend to include mainly those with complex needs and/or specific disease conditions. This study captured the service gap where these non-frail older patients might have no specific medical problem upon discharge but they might encounter residual health and social issues when returning home.

**Methods:**

Discharged community-dwelling non-frail older adults from an emergency medical ward were recruited and randomized into either intervention (*n* = 37) or control (*n* = 38) group. The intervention group received a 12-week complex interventions that included structured assessment, health education, goal empowerment, and care coordination supported by a health-social team. The control group received usual discharge care and monthly social call. The primary outcome was health-related quality of life (HRQoL). Secondary outcomes included activities of daily living (ADL), the presence of depressive symptoms, and the use of health services. The outcomes were measured at pre-intervention (T1) and at three months post-intervention (T2). The independent t-test or the Mann-Whitney U test was used to analyze the group differences in HRQoL, ADL, and presence of depressive symptoms according to the normality of data.

**Results:**

Analysis showed that the intervention group experienced a statistically significantly improvement in the mental component scale of quality of life (*p* = .036), activities of daily living (*p* = .005), and presence of depressive symptoms (*p* = .035) at T2 compared with at T1. No significant differences were found in the control group.

**Conclusions:**

Supporting self-care is necessary to enable community-dwelling non-frail older adults to be independent to the fullest extent possible in the community. The promising results found in this pilot study suggested that the integration of the health-social partnership into transitional care practice is effective and can be sustained in the community. Future studies can draw on these findings and maximize the integrated care quality during the transition phase.

**Trial registration:**

NCT04434742 (date: 17 June 2020, retrospectively registered).

## Background

Transitioning from hospital to home is often a vulnerable time for older adults. While evidence has shown that many evidence-based care models such as transitional care programs and discharge planning programs are effective at reducing readmission rates and improving health after patients return home [1, 2], their target groups are often frail older adults who are described as having multiple chronic diseases, less multisystem reserve capacity, and dependency in function [3]. For instance, a recent review concluded that although the health status criteria for participation varied among the reviewed studies, all used a screening tool that put participants at risk for increased health service utilization, such as needing assistance with activities of daily living, complex health conditions, and high frailty scores [4]. This is understandable because frail older adults have a high risk of readmission and require intensive health care support to remain in the community after hospital discharge [5]. Older adults who are medically fit for discharge and functionally independent are rarely the focus of attention. However, these non-frail older adults account for more than 85% of the aging population [6]. When they are discharged from hospital after acute illnesses, they face similar health and social problems as frail older adults, such as being physically unstable, having difficulty in managing prescribed therapeutic regimens, and having poor knowledge of available health care and social support services. In addition, older adults generally lose 5% of muscle strength per day of treatment while staying in a hospital bed, which leads to a loss of physical functioning and a reduction in self-care ability [7]. One study showed that the likelihood of transitioning from a state of lesser frailty to a state of greater frailty increased by approximately 40% when older adults were hospitalized [8]. In other words, pre-hospitalized non-frail patients may be deconditioned while hospitalized. When discharged, non-frail older adults need attention to help them resume normal life, stabilize their stay in the community, and prevent the possible decline of functional status.

Previous studies have shown that some hospital-based transitional care or discharge planning programs provide a health-social team to support older adults immediately after discharge. However, the results showed that their health and social care needs remain unmet due to the fragmentation of services and the diversity in intervention objectives provided by the health care team and social workers [9]. The World Health Organization World Alliance for Patient Safety reiterated that improving coordination and communication among different professions should be the first priority for providing quality of care to patients [10]. Some developed countries, such as the United Kingdom and Sweden, have recognized the importance of the health-social partnership and integrated it into policy [11], though results reported in the literature have shown that professionals in these countries are often unaware of their roles and responsibilities and the best evidence they can draw on when working with other disciplines [10, 12]. A recent review showed that although some studies used the term ‘collaborative care’ in the transitional care program, they failed to develop a well-structured model to help different disciplines work together [13].

This study endeavors to build a community-based health-social partnership program and subject it to empirical testing to support non-frail older adults in living to their fullest with optimum quality of life in their own environment after hospital discharge. We hypothesize that a properly functioning health-social team that provides comprehensive assessment in a home-based environment, strengthens self-care ability, and furnishes available community resources might improve quality of life among non-frail community-dwelling older adults. If proven successful, this model can be sustained in the community and bring us a step closer to the goal of successful aging in place.

## Methods

### Model of care

The community-based health-social partnership (CHSP) program is built on two main conceptual guides: Supported self-care and the Omaha System. The concept of supported self-care references the UK framework for promoting health and social partnership, which aims at supporting people in the community with long-term conditions [14]. At the delivery end, individualized case management was employed to support patients with enhanced knowledge, skills and confidence in caring for themselves. Efforts were directed at building a community-based intervention with a health-social partnership to ensure that system resources and collaboration among stakeholders could be activated to provide support to individuals when needed. There was a case manager to ensure that the support for individual clients in enhancing self-care was comprehensive and properly coordinated. The nurse, backed up by a multidisciplinary team, assumed the role of case manager in the program, and home visits and telephone calls were the two approaches to care delivery [15, 16]. The Omaha System was used as a comprehensive assessment-intervention-evaluation framework in this study to provide the skeleton for translating the concept of supported self-care. The Omaha System has been widely used in community health settings [17]. It has a very clear and comprehensive description of the assessment and intervention schemes. The clear description of the schemes facilitates the classification of the problems and the nature of the required intervention, thus leading to effective inter-professional collaboration.

### Study design, setting, and participants

This was a single-blind, randomized controlled trial. Subject recruitment took place in the emergency medical ward (EMW) of a hospital in Hong Kong. The EMW is an extension of the Accident and Emergency service, with a total of 36 beds. Patients were included if they resided in the service areas of the study hospital, were aged 60 or over, were cognitively competent with a score greater than 26 in the Montreal Cognitive Assessment Hong Kong version [18], were living at home before and after discharge from the EMW, had scores of < 5 on the Clinical Frailty Scale (Note: a patient is considered to be non-frail if they have a score less than 5) [19], and were fit for medical discharge. Patients who were not able to communicate, could not be reached by phone, were bed-bound, had active psychiatric problems, were already engaged in other structured health or social programs, and would not be staying in Hong Kong for the three months of the study were excluded. A research assistant introduced the program including its aim, benefits, and the potential risks of participating to eligible patients. Patients who agreed to participate would sign a consent form. Ethical approval was obtained from the ethics committee of the hospital before data collection (REC No. 17–0015/FR-3). The reporting of this trial follows the CONSORT guideline (www.consort-statement.org).

### Procedures

A research team member who was not involved in the subject recruitment and data collection generated a random assignment schedule using the software Research Randomizer. The assignment of groups was put in sealed envelopes. The research team member, upon successfully recruiting a subject, opened an envelope in sequence after the enrolled subjects had finished the baseline assessment, and allocated the subject to the assigned intervention or control group. In this study, the research assistant who collected the data was blinded but the patients and provider involved in the intervention were not.

### Sample size

Power calculations were not conducted for the pilot study [20], because the data collected from the pilot study will be used to inform sample size estimation and power analysis for a future large-scale study. A systematic review [21], however, found that a general rule for pilot studies is to take 30 patients per arm. With reference to the 10% attrition reported in previous programs for community-dwelling older adults [21], a sample size of 66 patients was used in this study.

### Intervention group

This is a three-month health-social partnership program. The first month acted as a loading dose, where more intensive support was provided to older adults post-discharge. The second and third months were considered as a maintenance dose, representing the continuation of the intervention to sustain the therapeutic effect.

After each client was admitted to the EMW, an advanced practice nurse (APN) from a hospital discharge team visited them to familiarize him/herself with their condition and prepare a discharge plan. A face-to-face or telephone call handover between the APN and the project nurse case manager (NCM) was performed before the client was discharged. The past and current medical conditions, medical and nursing management, and follow-up appointments were discussed. After discharge home, the NCM, functioning as the leader of health-social care team, conducted the initial assessment in the first home visit to identify the client’s health and social problems within one week of discharge. Community workers, supervised by both the nurse case manager and social worker, provided telephone follow-up and subsequent home visits to monitor the client’s progress and provide support when necessary.

The Omaha System was used by the NCM in the first home visit because it provides categories to connect the home health care problems of post-discharged non-frail older adults to the related nursing interventions. According to the problems identified, the NCM provided interventions in accordance with the Omaha System scheme, which included health teaching, guidance and counseling, treatment and procedures, case management and surveillance. The NCM also coordinated care across a range of settings, from the home to the community center or hospital when necessary. In order to provide better care to clients, interdisciplinary case conferences were held regularly between the NCM and social workers, with the involvement of the APN if appropriate. During the conference, the health-social team members communicated each other’s role in managing the case, which increased understanding and collaboration in the process. In addition, events such as the progress and concerns of clients, and suggestions for further actions, modifications, or adjustment of interventions were reviewed.

### Control group

The control group received usual discharge care and community resources that were made available to them as appropriate. A monthly social call was made to each client in the control group in order to exclude social effects. The social call was provided by a research team member who was not involved in data collection. The contents of the social call, such as asking about entertainment and clients’ hobbies, were set in the protocol.

### Outcome measures

Data were collected at two time intervals—at baseline pre-intervention (T1) and at three months when the interventions were completed – to determine the effects of the study (T2). Health-related quality of life (HRQoL) was used as the primary outcome measure of the study. The goal for this program was to enable older adults to live with optimum quality of life in their own environment through receiving support from the collaboration of nurse case managers and social workers. Quality of life was measured by SF-12, which has been shown to be useful in Chinese elderly patients [22]. The questionnaire has 12 items organized into eight categories (physical functioning, role limitation due to emotional and physical problems, mental health scale, general health, bodily pain, social functioning, and vitality), and has been validated in numerous studies [23]. The Cronbach’s alpha coefficient was 0.7 [24].

Secondary outcomes included activities of daily living (ADL), the presence of depressive symptoms, and the use of health services. ADL was measured by the Chinese version of the Modified Barthel Index [25]. The presence of depressive symptoms was measured by the Geriatric Depression Scale [26]. Good validity and reliability were shown in these two measuring scales among the Chinese elderly population [25–27].

The outcome of health service use included the total number of unplanned outpatient department, general practitioner, and emergency department visits, hospital admissions and total number of health service attendances. This information was collected from the subjective reports of participants. They were asked about the number of attendances within the last three months prior to both T1 and T2 data collection.

### Statistical analysis

Statistical tests were performed using the Statistical Package for Social Sciences (SPSS) version 23 software. The principles of intention-to-treat analysis were followed. Descriptive analyses were used for describing the baseline demographic data. The independent t-test or the Mann-Whitney U test analyzed the group differences in HRQoL, ADL, and presence of depressive symptoms according to the normality of data. The time differences between T1 and T2 were analyzed using the paired t-test or Wilcoxon’s signed rank test. Logistic regression and Chi-squared tests were used to analyze health service use in dichotomized outcomes (i.e. health service use and no unplanned service use). Adjusted and unadjusted models were performed in all cases where regression modelling was done. Variables including sex, age, household composition, and financial status were adjusted, since they are likely to affect outcomes [28]. Odds ratios (OR) with 95% confidence intervals (CI) were calculated and reported. A multiple imputation procedure was employed to impute the missing data [29].

## Results

### Participants flow

**Fig. 1:**
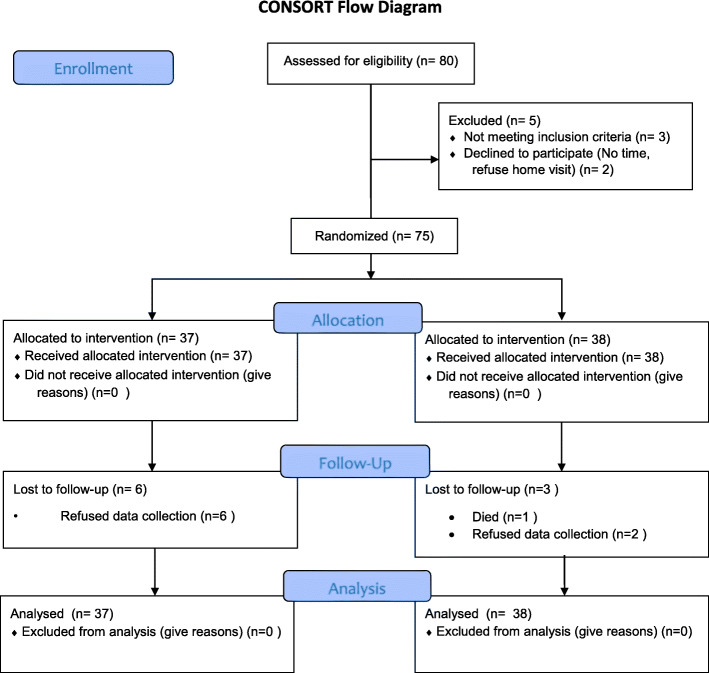
CONSORT table

Of the 80 potential discharged community-dwelling older adults who were assessed for eligibility, 75 eligible participants agreed to join the program and were randomized into intervention (*n* = 37) or control groups (*n* = 38). During the 12-week program, six participants (16.2%) in the intervention group refused T2 data collection because of time constraints. Similarly, three participants (7.9%) in the control group withdrew due to death and data collection refusal (Fig. 1).

### Sample description

In general, the baseline characteristics and outcomes of both groups were well balanced, with no statistically significant differences. The mean age for intervention and control groups was 79.0 and 80.3, respectively. The three most common chronic diseases were hypertension (81.3%), pain (58.7%), and diabetes mellitus (40.0%). The baseline demographic characteristics and outcome scores are reported in Tables 1 and 2, respectively.

**Table 1:** Baseline demographic characteristics

		Total		Intervention		Control		
		Count	%	Count	%	Count	%	*p*-value.
Gender	Male	34	45.30%	20	54.10%	14	36.80%	.13^a^
	Female	41	54.70%	17	45.90%	24	63.20%	
	Total	75	100.00%	37	100.00%	38	100.00%	
Marital status	Single	6	8.00%	3	8.10%	3	7.90%	.25^a^
	Married	28	37.30%	15	40.50%	13	34.20%	
	Divorced	3	4.00%	3	8.10%	0	0.00%	
	Widowed	38	50.70%	16	43.20%	22	57.90%	
	Total	75	100.00%	37	100.00%	38	100.00%	
Education level	No formal education	20	26.70%	8	21.60%	12	31.60%	.40^a^
	Primary	30	40.00%	14	37.80%	16	42.10%	
	Secondary	20	26.70%	11	29.70%	9	23.70%	
	Tertiary	5	6.70%	4	10.80%	1	2.60%	
	Others	0	0.00%	0	0.00%	0	0.00%	
	Total	75	100.00%	37	100.00%	38	100.00%	
Pain	No	31	41.30%	16	43.20%	15	39.50%	.74^a^
	Yes	44	58.70%	21	56.80%	23	60.50%	
	Total	75	100.00%	37	100.00%	38	100.00%	
Hypertension	No	14	18.70%	6	16.20%	8	21.10%	.59^a^
	Yes	61	81.30%	31	83.80%	30	78.90%	
	Total	75	100.00%	37	100.00%	38	100.00%	
Diabetes mellitus	No	45	60.00%	19	51.40%	26	68.40%	.13^a^
	Yes	30	40.00%	18	48.60%	12	31.60%	
	Total	75	100.00%	37	100.00%	38	100.00%	
Stroke	No	68	90.70%	36	97.30%	32	84.20%	.11^b^
	Yes	7	9.30%	1	2.70%	6	15.80%	
	Total	75	100.00%	37	100.00%	38	100.00%	
Cancer	No	69	92.00%	33	89.20%	36	94.70%	.43^b^
	Yes	6	8.00%	4	10.80%	2	5.30%	
	Total	75	100.00%	37	100.00%	38	100.00%	
Arthritis	No	70	93.30%	33	89.20%	37	97.40%	.20^b^
	Yes	5	6.70%	4	10.80%	1	2.60%	
	Total	75	100.00%	37	100.00%	38	100.00%	
Depression	No	73	97.30%	36	97.30%	37	97.40%	1.00^b^
	Yes	2	2.70%	1	2.70%	1	2.60%	
	Total	75	100.00%	37	100.00%	38	100.00%	

a = Pearson Chi-square test; b = Fisher’s exact test; *p < .05

**Table 2:** Baseline primary and secondary outcome measures

		Total (*n* = 75)		Intervention group (n = 37)		Control group (n = 38)		*t*-test
		Mean	(*SD*)	Mean	(*SD*)	Mean	(*SD*)	*p*-value
HRQoL	PCS	36.9	(10.6)	36.0	(10.2)	37.8	(11.0)	.47
	MCS	51.9	(11.7)	51.9	(12.5)	52.0	(11.0)	.98
ADL		97.8	(4.33)	97.8	(3.93)	97.9	(4.73)	.41
Depressive symptoms		4.08	(3.00)	4.16	(3.80)	4.84	(4.37)	.53
Use of health services	GOPD visits	0.23	(0.53)	0.30	(0.62)	0.16	(0.44)	.31
	GP visits	1.20	(1.89)	1.16	(1.74)	1.24	(2.05)	.84
	ED visits	1.36	(0.73)	1.43	(0.87)	1.29	(0.57)	.62
	Hospital admissions	1.31	(0.61)	1.30	(0.66)	1.32	(0.57)	.76
	Total number of health service attendances	2.79	(2.29)	2.89	(2.50)	2.68	(2.09)	.87

**Note:** *SD* = *standard deviation*; HRQoL = health-related quality of life; PCS = physical component score; MCS = mental component score; ADL = activities of daily living; GDS = Geriatric Depression Scale; GOPD = general out-patient department; GP = general practitioner; ED = emergency department; *p-value < .05

### Impact of the interventions on outcomes

Table 3 illustrates the changes in the mean scores for HRQoL, ADL, and depressive symptoms of the two groups at T1 and T2. There was a significant time effect between T1 and T2 (*p* = .036) in the intervention group in terms of the mental component scale of quality of life. However, no significant differences were found between the intervention and control groups in either the physical (*p* = .54) or mental (*p* = .99) components of quality of life. Similar results were demonstrated in ADL and depressive symptoms. Statistically significant improvements were observed in the intervention group for both ADL (*p* = .005) and depressive symptoms (*p* = .035), though there was no significant time effect in the control group and no between-group effect for either outcome (Table 3). As seen in Table 4, both adjusted and unadjusted analysis showed that there were no statistically significant between-group and within-group differences in use of health services.

**Table 3:** HRQoL, ADL, and depressive symptoms according to group before and after the intervention

		IG		CG		Within-group analysis		T2 between-group analysis
		T1	T2	T1	T2	IG	CG	
		Mean ± SD				*p*-value		
HRQoL^a^	PCS	36.0 ± 10.2	41.0 ±9.35	37.8±11.0	39.5±10.6	.071	.48	.54
	MCS	51.9 ± 12.5	55.8 ± 10.9	52.0 ± 11.0	56.3 ± 10.9	.036*	.054	.99
ADL^b^		97.8 ± 3.93	99.2 ± 2.90	97.9 ± 4.73	98.6 ± 4.04	.005*	.68	.56
Depressive symptoms^c^		4.16 ± 3.80	3.23 ± 4.10	4.84 ± 4.37	3.77 ± 4.12	.035*	.16	.54

**Note:** *SD* = *standard deviation*; HRQoL = health-related quality of life; PCS = physical component score; MCS = mental component score; ADL = activities of daily living; IG = intervention group; CG = control group; *p-value < .05

a Higher scores indicate better quality of life

b Higher scores indicate better activity of daily living

c Higher scores indicate higher severity of depressive symptoms

**Table 4:** Adjusted and unadjusted odds of health services use at T2, intervention vs control groups

Use of Health Services	Unadjusted				Adjusted^			
	(95% CI)				(95% CI)			
	OR	Lower	Upper	*p*-value	OR	Lower	Upper	*p*-value
GOPD visits	1.08	(0.29	3.95)	0.91	0.75	(0.14	4.04)	0.74
GP visits	1.58	(0.58	4.31)	0.38	2.47	(0.65	9.32)	0.18
ED visits	1.23	(0.40	3.84)	0.72	0.88	(0.25	3.10)	0.85
Hospital admission	1.23	(0.40	3.84)	0.72	0.88	(0.25	3.10)	0.85
Total times of health service attendances	1.51	(0.56	4.06)	0.41	1.66	(0.51	5.36)	0.40

**Note:** CI = confidence interval; OR = odds ratios; GOPD = general out-patient department; GP = general practitioner; ED = emergency department; *p-value < .05; ^ adjusted for sex, age, household composition, financial status

## Discussion

Studies of transitional care programs tend to include subjects mainly with complex needs and/ or specific disease conditions. To our knowledge, this community-based health-social partnership program is one of few that capture the service gap where non-frail older patients may have no specific medical problem upon discharge but encounter residual health and social issues when returning home. Our findings demonstrated that post-intervention improvement was significantly better for clients in the intervention group with regard to their mental component scale of quality of life, ADL, and depressive level. The results are preliminary, but could be considered meaningful given the impact of these outcomes to the determination of healthy living for community-dwelling older adults.

Previous studies supporting discharged patients were largely designed and delivered by a hospital-based health care professional team, such as geriatricians, nurses, occupational therapists, and physiotherapists. Although evidence has shown that these hospital-based discharge planning and transitional care programs are effective at reducing hospitalization and improving health after patients return home, these programs tend to be health focused and confine to a time period immediately after discharge to stabilize the patients physically in the community. However, given the long-term aim of keeping discharged older patients able to live independently in the community with optimal health and reduce the use of hospital services, responsibility for their health should lie with the community itself, not the hospitals. The centerpiece of the present study was the role of the NCM, who helped form a link among hospital health service providers, community social agencies, and patients during the transitional period. On one hand, the NCM visited the hospital on a weekly basis to discuss patients’ medical conditions with the hospital staff and facilitate the formulation of a consistent, appropriate, and coordinated care plan in a timely fashion related to discharge. On the other hand, the NCM made use of available resources from community social agencies to support a safe, healthy, and sustainable living environment for patients after discharge. The NCM in the program acts as a link interfacing hospital and community care, reducing the information loss during handover and improving the continuation of treatment, which smooths the transition processes and facilitates the sustainability of the program.

Literature has shown that a lack of role clarity for each provider in a multidisciplinary team, time constraints, and inadequate team meetings are often the reasons for disjointed hospital-community care transitions and high rates of adverse medical events and poor HRQoL in discharged patients [30]. An important contribution of this study is the building of a framework that guides the inter-professional team to work effectively and efficiently with both patients and other team members. Specifically, at the patient level, individualized case management was used to enhance patients’ skills, knowledge, and confidence in caring for themselves. At the team level, an embedded system that linked health, social care, and patients was built to effect individual change, including care protocols that quantified and recognized the role of each provider and defined appropriate agents of care and collaborators, referral protocols, multidisciplinary access to care plans and care outcomes, rules and protocols governing information sharing, and regular team communication and meetings. The positive results found in our study indicate that this approach had the desired effect. As such, future studies can draw on these findings and maximize the integrated care quality and overall team performance during the transition phase. With this pilot study suggesting some positive outcomes of the program, a main study will be launched. The program was quite human intensive with home visits by NCM. The main study can consider employing e-health applications to reduce some human resources, and test the cost-effectiveness of the program.

Although the effects of the current program were evident, several study limitations should be considered when interpreting the study findings. The small sample size recruited in the study due to its preliminary nature limited its statistical power to determine group differences in outcomes, although the findings do provide some insight into the effect of this program. In addition, since the follow-up period was 12 weeks, the sustainability effect after 12 weeks is unknown.

## Conclusion

The results of this pilot RCT suggest that the HRQoL, ADL, and depressive symptoms of non-frail older patients improved after receiving the community-based health-social partnership program. These encouraging results need to be confirmed by a large-scale interventional program that not only tests the sustainability of its effects, but also evaluates the extent to which the program might influence the quality of the inter-professional team process and performance.

## Data Availability

The datasets used and/or analyzed during the current study are available from the corresponding author on reasonable request.

## Abbreviations

HRQoL: Health-related quality of life
ADL: Activities of daily living
CHSP: The community-based health-social partnership
EMW: Emergency medical ward
APN: Advanced practice nurse
NCM: Nurse case manager
SF-12: Short-form-12 Health Survey
OR: Odd ratio
CI: Confidence interval
